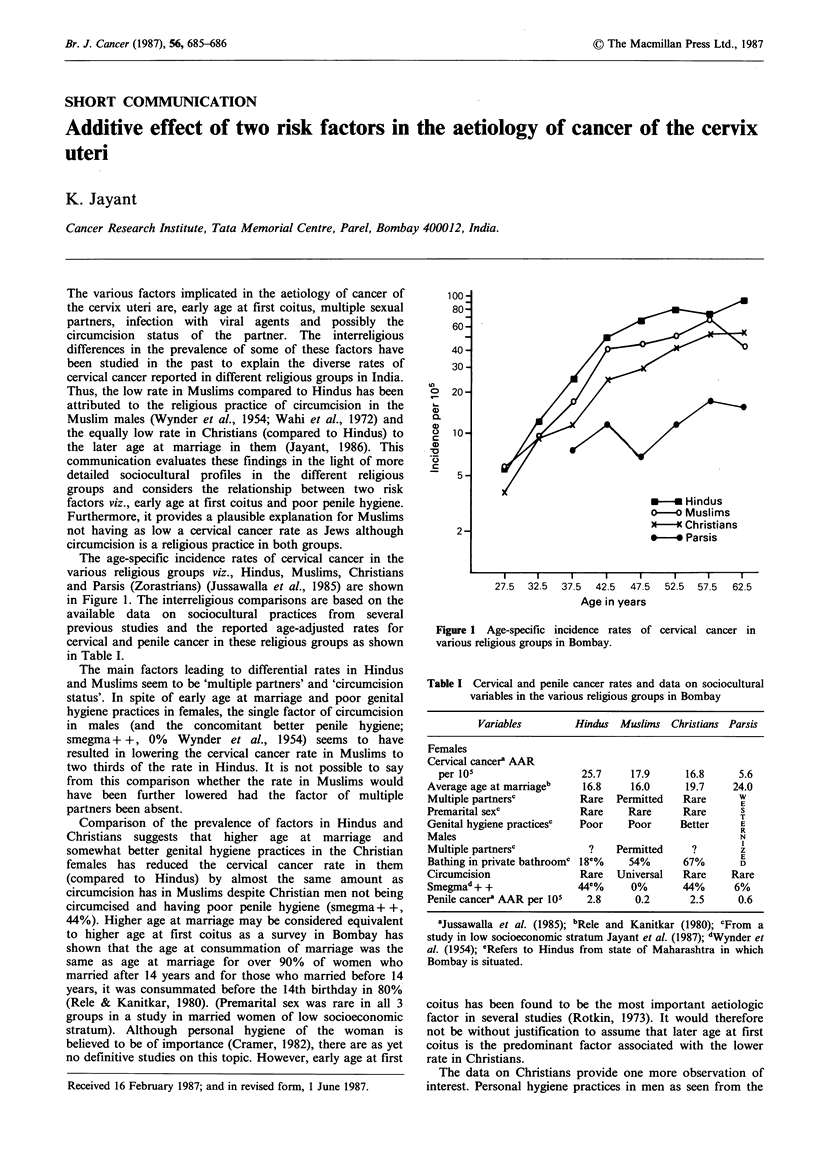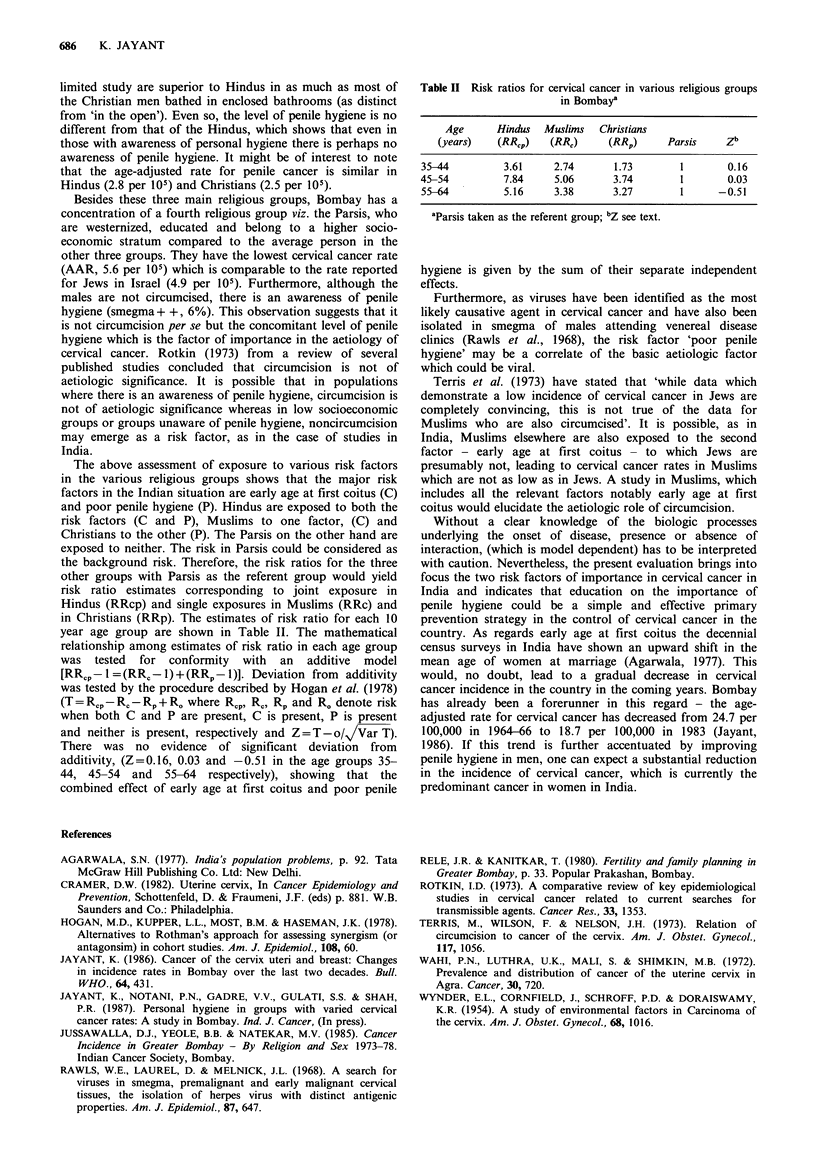# Additive effect of two risk factors in the aetiology of cancer of the cervix uteri.

**DOI:** 10.1038/bjc.1987.266

**Published:** 1987-11

**Authors:** K. Jayant

**Affiliations:** Cancer Research Institute, Tata Memorial Centre, Parel, Bombay, India.


Br. J. Cancer (1987), **56**, 685–686 © The Macmillan Press Ltd., 1987

SHORT COMMUNICATION

### Additive effect of two risk factors in the aetiology of cancer of the cervix uteri

K. Jayant

*Cancer Research Institute, Tata Memorial Centre, Parel, Bombay 400012, India.*

The various factors implicated in the aetiology of cancer of the cervix uteri are, early age at first coitus, multiple sexual partners, infection with viral agents and possibly the circumcision status of the partner. The interreligious differences in the prevalence of some of these factors have been studied in the past to explain the diverse rates of cervical cancer reported in different religious groups in India. Thus, the low rate in Muslims compared to Hindus has been attributed to the religious practice of circumcision in the Muslim males (Wynder *et al.*, 1954; Wahi *et al.*, 1972) and the equally low rate in Christians (compared to Hindus) to the later age at marriage in them (Jayant, 1986). This communication evaluates these findings in the light of more detailed sociocultural profiles in the different religious groups and considers the relationship between two risk factors *viz.*, early age at first coitus and poor penile hygiene. Furthermore, it provides a plausible explanation for Muslims not having as low a cervical cancer rate as Jews although circumcision is a religious practice in both groups.

The age-specific incidence rates of cervical cancer in the various religious groups *viz.*, Hindus, Muslims, Christians and Parsis (Zorastrians) (Jussawalla *et al.*, 1985) are shown in Figure 1. The interreligious comparisons are based on the available data on sociocultural practices from several previous studies and the reported age-adjusted rates for cervical and penile cancer in these religious groups as shown in Table I.

The main factors leading to differential rates in Hindus and Muslims seem to be 'multiple partners' and 'circumcision status'. In spite of early age at marriage and poor genital hygiene practices in females, the single factor of circumcision in males (and the concomitant better penile hygiene; smegma++, 0% Wynder *et al.*, 1954) seems to have resulted in lowering the cervical cancer rate in Muslims to two thirds of the rate in Hindus. It is not possible to say from this comparison whether the rate in Muslims would have been further lowered had the factor of multiple partners been absent.

Comparison of the prevalence of factors in Hindus and Christians suggests that higher age at marriage and somewhat better genital hygiene practices in the Christian females has reduced the cervical cancer rate in them (compared to Hindus) by almost the same amount as circumcision has in Muslims despite Christian men not being circumcised and having poor penile hygiene (smegma++, 44%). Higher age at marriage may be considered equivalent to higher age at first coitus as a survey in Bombay has shown that the age at consummation of marriage was the same as age at marriage for over 90% of women who married after 14 years and for those who married before 14 years, it was consummated before the 14th birthday in 80% (Rele & Kanitkar, 1980). (Premarital sex was rare in all 3 groups in a study in married women of low socioeconomic stratum). Although personal hygiene of the woman is believed to be of importance (Cramer, 1982), there are as yet no definitive studies on this topic. However, early age at first coitus has been found to be the most important aetiologic factor in several studies (Rotkin, 1973). It would therefore not be without justification to assume that later age at first coitus is the predominant factor associated with the lower rate in Christians.

The data on Christians provide one more observation of interest. Personal hygiene practices in men as seen from the

**Figure 1** Age-specific incidence rates of cervical cancer in various religious groups in Bombay.

**Table I** Cervical and penile cancer rates and data on sociocultural variables in the various religious groups in Bombay

| *Variables* | *Hindus* | *Muslims* | *Christians* | *Parsis* |
|---|---|---|---|---|
| Females | | | | |
| Cervical cancer[a] AAR per $10^5$ | 25.7 | 17.9 | 16.8 | 5.6 |
| Average age at marriage[b] | 16.8 | 16.0 | 19.7 | 24.0 |
| Multiple partners[c] | Rare | Permitted | Rare | WESTERNIZED |
| Premarital sex[c] | Rare | Rare | Rare | |
| Genital hygiene practices[c] | Poor | Poor | Better | |
| Males | | | | |
| Multiple partners[c] | ? | Permitted | ? | |
| Bathing in private bathroom[c] | 18[e]% | 54% | 67% | |
| Circumcision | Rare | Universal | Rare | Rare |
| Smegma[d]++ | 44[e]% | 0% | 44% | 6% |
| Penile cancer[a] AAR per $10^5$ | 2.8 | 0.2 | 2.5 | 0.6 |

[a]Jussawalla *et al.* (1985); [b]Rele and Kanitkar (1980); [c]From a study in low socioeconomic stratum Jayant *et al.* (1987); [d]Wynder *et al.* (1954); [e]Refers to Hindus from state of Maharashtra in which Bombay is situated.

Received 16 February 1987; and in revised form, 1 June 1987.

limited study are superior to Hindus in as much as most of the Christian men bathed in enclosed bathrooms (as distinct from 'in the open'). Even so, the level of penile hygiene is no different from that of the Hindus, which shows that even in those with awareness of personal hygiene there is perhaps no awareness of penile hygiene. It might be of interest to note that the age-adjusted rate for penile cancer is similar in Hindus (2.8 per $10^5$) and Christians (2.5 per $10^5$).

Besides these three main religious groups, Bombay has a concentration of a fourth religious group *viz.* the Parsis, who are westernized, educated and belong to a higher socioeconomic stratum compared to the average person in the other three groups. They have the lowest cervical cancer rate (AAR, 5.6 per $10^5$) which is comparable to the rate reported for Jews in Israel (4.9 per $10^5$). Furthermore, although the males are not circumcised, there is an awareness of penile hygiene (smegma++, 6%). This observation suggests that it is not circumcision *per se* but the concomitant level of penile hygiene which is the factor of importance in the aetiology of cervical cancer. Rotkin (1973) from a review of several published studies concluded that circumcision is not of aetiologic significance. It is possible that in populations where there is an awareness of penile hygiene, circumcision is not of aetiologic significance whereas in low socioeconomic groups or groups unaware of penile hygiene, noncircumcision may emerge as a risk factor, as in the case of studies in India.

The above assessment of exposure to various risk factors in the various religious groups shows that the major risk factors in the Indian situation are early age at first coitus (C) and poor penile hygiene (P). Hindus are exposed to both the risk factors (C and P), Muslims to one factor, (C) and Christians to the other (P). The Parsis on the other hand are exposed to neither. The risk in Parsis could be considered as the background risk. Therefore, the risk ratios for the three other groups with Parsis as the referent group would yield risk ratio estimates corresponding to joint exposure in Hindus (RRcp) and single exposures in Muslims (RRc) and in Christians (RRp). The estimates of risk ratio for each 10 year age group are shown in Table II. The mathematical relationship among estimates of risk ratio in each age group was tested for conformity with an additive model $[RR_{cp}-1=(RR_c-1)+(RR_p-1)]$. Deviation from additivity was tested by the procedure described by Hogan *et al.* (1978) $(T=R_{cp}-R_c-R_p+R_o$ where $R_{cp}$, $R_c$, $R_p$ and $R_o$ denote risk when both C and P are present, C is present, P is present and neither is present, respectively and $Z=T-o/\sqrt{\text{Var T}})$. There was no evidence of significant deviation from additivity, (Z=0.16, 0.03 and −0.51 in the age groups 35–44, 45–54 and 55–64 respectively), showing that the combined effect of early age at first coitus and poor penile hygiene is given by the sum of their separate independent effects.

**Table II** Risk ratios for cervical cancer in various religious groups in Bombay[a]

| *Age (years)* | *Hindus ($RR_{cp}$)* | *Muslims ($RR_c$)* | *Christians ($RR_p$)* | *Parsis* | *Z*[b] |
|---|---|---|---|---|---|
| 35–44 | 3.61 | 2.74 | 1.73 | 1 | 0.16 |
| 45–54 | 7.84 | 5.06 | 3.74 | 1 | 0.03 |
| 55–64 | 5.16 | 3.38 | 3.27 | 1 | −0.51 |

[a]Parsis taken as the referent group; [b]Z see text.

Furthermore, as viruses have been identified as the most likely causative agent in cervical cancer and have also been isolated in smegma of males attending venereal disease clinics (Rawls *et al.*, 1968), the risk factor 'poor penile hygiene' may be a correlate of the basic aetiologic factor which could be viral.

Terris *et al.* (1973) have stated that 'while data which demonstrate a low incidence of cervical cancer in Jews are completely convincing, this is not true of the data for Muslims who are also circumcised'. It is possible, as in India, Muslims elsewhere are also exposed to the second factor – early age at first coitus – to which Jews are presumably not, leading to cervical cancer rates in Muslims which are not as low as in Jews. A study in Muslims, which includes all the relevant factors notably early age at first coitus would elucidate the aetiologic role of circumcision.

Without a clear knowledge of the biologic processes underlying the onset of disease, presence or absence of interaction, (which is model dependent) has to be interpreted with caution. Nevertheless, the present evaluation brings into focus the two risk factors of importance in cervical cancer in India and indicates that education on the importance of penile hygiene could be a simple and effective primary prevention strategy in the control of cervical cancer in the country. As regards early age at first coitus the decennial census surveys in India have shown an upward shift in the mean age of women at marriage (Agarwala, 1977). This would, no doubt, lead to a gradual decrease in cervical cancer incidence in the country in the coming years. Bombay has already been a forerunner in this regard – the age-adjusted rate for cervical cancer has decreased from 24.7 per 100,000 in 1964–66 to 18.7 per 100,000 in 1983 (Jayant, 1986). If this trend is further accentuated by improving penile hygiene in men, one can expect a substantial reduction in the incidence of cervical cancer, which is currently the predominant cancer in women in India.

### References

AGARWALA, S.N. (1977). *India's population problems*, p. 92. Tata McGraw Hill Publishing Co. Ltd: New Delhi.

CRAMER, D.W. (1982). Uterine cervix, In *Cancer Epidemiology and Prevention*, Schottenfeld, D. & Fraumeni, J.F. (eds) p. 881. W.B. Saunders and Co.: Philadelphia.

HOGAN, M.D., KUPPER, L.L., MOST, B.M. & HASEMAN, J.K. (1978). Alternatives to Rothman's approach for assessing synergism (or antagonsim) in cohort studies. *Am. J. Epidemiol.*, **108**, 60.

JAYANT, K. (1986). Cancer of the cervix uteri and breast: Changes in incidence rates in Bombay over the last two decades. *Bull. WHO.*, **64**, 431.

JAYANT, K., NOTANI, P.N., GADRE, V.V., GULATI, S.S. & SHAH, P.R. (1987). Personal hygiene in groups with varied cervical cancer rates: A study in Bombay. *Ind. J. Cancer*, (In press).

JUSSAWALLA, D.J., YEOLE, B.B. & NATEKAR, M.V. (1985). *Cancer Incidence in Greater Bombay – By Religion and Sex* 1973–78. Indian Cancer Society, Bombay.

RAWLS, W.E., LAUREL, D. & MELNICK, J.L. (1968). A search for viruses in smegma, premalignant and early malignant cervical tissues, the isolation of herpes virus with distinct antigenic properties. *Am. J. Epidemiol.*, **87**, 647.

RELE, J.R. & KANITKAR, T. (1980). *Fertility and family planning in Greater Bombay*, p. 33. Popular Prakashan, Bombay.

ROTKIN, I.D. (1973). A comparative review of key epidemiological studies in cervical cancer related to current searches for transmissible agents. *Cancer Res.*, **33**, 1353.

TERRIS, M., WILSON, F. & NELSON, J.H. (1973). Relation of circumcision to cancer of the cervix. *Am. J. Obstet. Gynecol.*, **117**, 1056.

WAHI, P.N., LUTHRA, U.K., MALI, S. & SHIMKIN, M.B. (1972). Prevalence and distribution of cancer of the uterine cervix in Agra. *Cancer*, **30**, 720.

WYNDER, E.L., CORNFIELD, J., SCHROFF, P.D. & DORAISWAMY, K.R. (1954). A study of environmental factors in Carcinoma of the cervix. *Am. J. Obstet. Gynecol.*, **68**, 1016.